# FlowCLOc, a New Tool for Selecting the Most Appropriate Antibodies in Flow Cytometry

**DOI:** 10.3390/ijms27041664

**Published:** 2026-02-09

**Authors:** Valentina Serra, Valeria Orrù, Sandra Lai, Mariano Dei, Michele Marongiu, Maria Grazia Piras, Francesca Virdis, Matteo Floris, Giuseppe Delogu, Valeria Lodde, Mauro Pala, Maristella Pitzalis, Edoardo Fiorillo, Francesco Cucca

**Affiliations:** 1 Institute for Genetic and Biomedical Research, National Research Council, 08045 Lanusei, Italy; 2 Institute for Genetic and Biomedical Research, National Research Council, 09042 Monserrato, Italy; 3 Department of Biomedical Sciences, University of Sassari, 07100 Sassari, Italy

**Keywords:** flow cytometry, PBMCs, frozen blood, cryopreservation, FlowCLOc

## Abstract

Circulating immune cells are frequently phenotyped by flow cytometry starting from frozen samples. However, cryopreservation can affect marker expression and cell recovery. To understand which antigens are detectable and reliable after sample cryopreservation, we compared 438 antibodies measured on B, T, and NK-enriched cells and monocytes in frozen lympho-monocytes and blood with the corresponding fresh blood samples. Cryopreservation affected the expression of 283 markers in lympho-monocytes and 262 in blood, modifying them by more than 20% with respect to fresh blood. Thus, it is essential to carefully evaluate antibody performance when working with frozen samples. To maximize the usability of our results, make them publicly accessible and ready to visualize, we created a catalogue of marker expression variability before and after freezing, namely FlowCLOc. This catalogue simplifies flow cytometry panel design, reducing time-consuming preliminary tests to select the most appropriate and specific markers for staining both frozen and fresh samples.

## 1. Introduction

Flow cytometry is a technology that allows the simultaneous and rapid analysis of multiple parameters on a cell. It discriminates between cell size and granularity, based on fluorescent and light-scatter properties. Furthermore, it simultaneously detects several extracellular and intracellular antigens, enabling deep characterization of cells in terms of function, activation, and maturation. Thus, flow cytometry has a wide range of applications from basic research to clinical diagnostics.

In recent years, deep immunophenotyping by flow cytometry has been a useful tool for identifying the genetic regulation of immune cell levels and for exploring its overlap with genetic signals associated with complex diseases [1,2,3]. Moreover, immunophenotyping is a useful approach for the longitudinal monitoring of human immune status [4,5] for a better understanding of the immune dysregulation occurring during senescence or in specific diseases [6]. Flow cytometry is also widely used in clinical practice, for example, to monitor chimeric antigen receptor T cell (CAR-T) therapy and to infuse cells in the tumor environment [7,8].

Fresh whole blood (fWB) is considered the gold standard for immunophenotyping by flow cytometry; however, its use could become infeasible when dealing with extensive and multicentric studies, or when the recruitment center is far from the flow cytometry facility. In these instances, sample cryopreservation is the solution to overcome these limitations. Nowadays, clinical trials and translational research are largely carried out through biobanks, in which millions of samples are collected and stored for subsequent use in multi-omic studies [9,10].

Cryopreserved whole blood (cWB) or lympho-monocytes are suitable samples for immunophenotyping by flow cytometry [11,12,13]. However, cryopreservation and sample manipulation could affect the detection of cell antigens: temperature shifts or media changes might induce the internalization of receptors expressed on the cell surface. For instance, chemokine receptors, which are involved in cell migration, are characterized by seven fluid transmembrane domains that move within the cell membrane. This flexibility is necessary for ligand–receptor binding, but it can reduce the detection of these proteins [14,15]. In such cases, staining at the physiological temperature of 37 °C can improve their binding with specific antibodies [5].

Additionally, the method used to cryopreserve whole blood or lympho-monocytes can affect cell viability as well as the expression of several antigens [13,16]. These and other issues can affect multicolor-panel design, particularly when it is necessary to characterize very rare cell populations or use a large number of fluorochromes simultaneously, as in spectral flow cytometry.

To the best of our knowledge, no previous work has systematically compared the expression of hundreds of surface markers on fresh and cryopreserved lymphocyte subsets. Some companies provide online tools to compare the expression of antibody clones on morphologically characterized leukocytes from fresh samples (lymphocytes, monocytes, and granulocytes), but they do not consider expression on specific cell subsets or on cells preserved under different conditions.

Overall, we tested 438 antibody clones, corresponding to 356 surface antigens, in fWB, cWB, and cryopreserved peripheral blood mononuclear cells (cPBMCs). We then created an interactive database, namely Flow cytometry CLOne comparison (FlowCLOc), to compare the variability in expression of each antigen measured on B, T, and Natural Killer-enriched (NKe) cells. This database allows researchers to select the most reliable antibodies to design a multicolor flow cytometry panel, depending on the available starting biological material.

## 2. Results

### 2.1. Preliminary Considerations

To cryopreserve samples, we used 10% DMSO concentration, which is the most widely used freezing method in laboratories for storing both blood and PBMC samples for subsequent immunophenotyping studies by flow cytometry [11,12]. The choice of this freezing procedure was also dictated by a comparison of five blood freezing methods with their corresponding fresh samples that we performed in a previous manuscript [13]. Of these five methods, the one used in the present study produced the most reliable results in terms of similarity to fresh blood. Furthermore, the same cryopreservation procedure (10% DMSO) was previously used to compare immune cell subsets measured in cryopreserved PBMCs and their corresponding fresh blood samples, obtaining a very high correlation between them [17].

**Figure 1:**
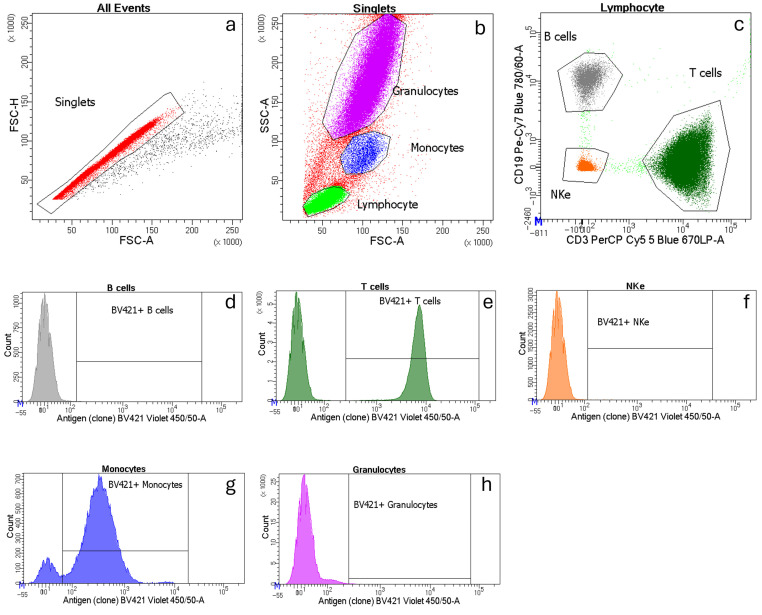
Representative example of a gating strategy in fresh blood. Dot plots showing: (**a**) single cells (red); (**b**) morphologically identified lymphocytes (light green), monocytes (blue), and granulocytes (violet); (**c**) T cells (dark green, CD3+ CD19−-), B cells (grey, CD3− CD19+), and NKe cells (orange, CD3− CD19−). Histograms represent (**d**) B cells, (**e**) T cells, (**f**) NKe cells, (**g**) monocytes, and (**h**) granulocytes; cells bound to a BV421-positive antibody (**e**,**g**) are shown on the right on the *x*-axis. The number of cells is indicated on the *y*-axis.

To evaluate whether cryopreservation affected the relative proportions of the main lymphocyte subsets, we compared the levels of cells and antigen expression in frozen samples (cWB or cPBMCs) with those in fresh blood. More in detail, we considered the variations that cryopreservation caused in terms of (i) relative frequency (with respect to the hierarchically higher cell population) of cells positive for the antigen tested and (ii) the expression levels of that antigen on the corresponding positive cell subset. We first gave an overview of the main lymphocyte subsets (B, T, and NKe cells) identified by CD3-PerCP-Cy5 and CD19-PE-Cy7 antibodies and measured in all samples tested (*n* = 139). A representative example of a gating strategy is reported in Figure 1.

We then focused on the 438 antibodies, all conjugated with the BV421 fluorochrome, evaluated in B, T, NKe cells, and monocytes, and tested in three samples. Notably, the three samples used to measure each marker are not necessarily the same for all antibodies tested, as it was not possible to draw blood from the same three individuals for the entire timeframe required to analyze the 438 clones. However, the blood samples used to test an antibody from fresh blood are the same used to measure the same antibody after freezing (both frozen blood and PBMCs). The cut-off for declaring a substantial change with respect to fresh blood samples was a variation (increase or decrease) higher than 20% of the frozen/fresh sample ratio (see Section 4 for details).

NK cells are a lymphocyte subset generally identified by CD56 and CD16 markers; however, we did not have the corresponding antibodies systematically assessed in each sample tested. We thus performed a preliminary experiment, considering 104 fresh blood samples stained with the 6-color TBNK kit (BD Biosciences), which allows us to discriminate T cells by CD3, B cells by CD19, and NK cells by CD16 and CD56 (Figure S1). We then compared the frequency of NK cells, identified by CD16 and CD56 antibodies, with the NKe cells obtained by excluding B and T cells from the total lymphocytes. We observed that they correlated at 99.9% (*p*-value = 6.86 × 10^−11^) with a mean frequency of 18.1% for NK cells and 18.39% for NKe cells with respect to total lymphocytes. Thus, we considered lymphocytes that are negative for both CD3 and CD19 as a good proxy to identify NK cells, calling them NK-enriched cells.

To evaluate the specificity of each antibody, we first stained fresh samples with backbone antibodies (anti-CD3 and anti-CD19) without using any specific antibody conjugated with BV421 fluorochrome. As shown in Table S1, the background fluorescence in the BV421 emission channel was very low. Then, we assessed the isotype controls corresponding to each antibody used (Table S2). If the expression of a marker was below the isotype emission, the binding was considered background and the marker negative, whereas if the expression was greater than the isotype emission, the binding was considered reliable. Only reliable markers were compared in fresh and frozen samples. We observed that the background was always very low for lymphocytes, whereas it increased in monocytes after cryopreservation, especially for mouse IgG2a and rat IgG2b isotypes.

Data from the 438 clones and the corresponding isotype controls are available in the FlowCLOc platform (http://flowcloc.irgb.cnr.it/, accessed on 6 February 2026). The platform allows us to visualize a specific antigen, clone, sample, staining temperature (room temperature or 37 °C), cell type (B, T, NKe cells, or monocytes), and condition (fresh or frozen cells). Furthermore, different antigens, clones, and samples can be shown simultaneously to be compared. The selected sample is represented by dot plots and histograms. A representative example of FlowCLOc use is available in the Supplementary Materials (Video S1).

### 2.2. Effect of Cryopreservation on Lymphocytes and Their Main Subsets

The frequency of viable lymphocytes was very similar in cPBMCs and cWB samples (99.3% vs. 98.4%, respectively) (Table S3, Figure S2).

We next evaluated how cryopreservation influenced the distribution of B, T, and NKe cell subsets in fresh and frozen samples.

**Figure 2:**
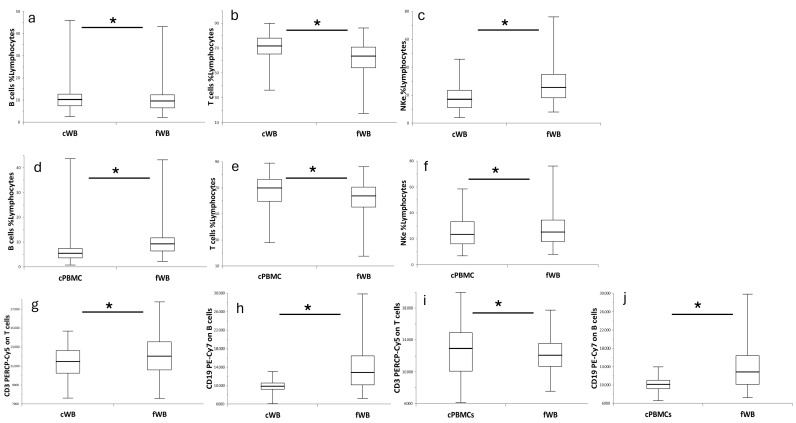
Comparison of T, B, and NKe cells in fresh and cryopreserved samples. Box plots show the distribution of cell frequencies (**a**–**f**) and protein expression levels (**g**–**j**) in frozen (cWB or cPBMCs) and fresh blood (fWB). Each box spans from quartile 1 to quartile 3; a line inside the box marks the median, whereas “whiskers” extend to the minimum and maximum. A paired *t*-test was applied to calculate *p*-values. The paired comparison of cPBMCs and fWB included 139 samples; whereas the comparison of cPBMCs and fWB comprises 126 paired samples. * = *p* < 0.05.

The proportions of both B and T cells increased by 17% and 22%, respectively, in cWB compared to fWB (*p*-values of 1.00 × 10^−2^ and 3.40 × 10^−19^, respectively), whereas NKe cell proportions were reduced by 33% (*p*-value of 5.54 × 10^−19^) (Figure 2a–c; Table S4). In cPBMCs, the proportion of B cells was reduced by 32% compared to fresh samples (*p* = 1.27 × 10^−21^), while the frequency of T cells increased by 16% (*p* = 4.03 × 10^−9^), and the proportion of NKe cells decreased by approximately 5% (*p* = 5.88 × 10^−3^) (Figure 2d–f).

The expression of CD3 and CD19, which characterize T and B cells, respectively, was reduced by 4% and 22% on cWB compared to fresh samples (*p*-values of 1.22 × 10^−5^ and 2.44 × 10^−16^, respectively; Figure 2g,h). In cPBMCs, a weak 8% increase in CD3 expression was observed (*p*-value = 4.10 × 10^−2^) alongside an 18% reduction in CD19 expression (*p*-value = 9.47 × 10^−13^) (Figure 2i,j).

### 2.3. Effect of Cryopreservation on B Cells

B cells are commonly known as antibody-producing cells, but along with their role in humoral immunity, they also generate cell-mediated immunity. Indeed, they are classified as professional antigen-presenting cells, like dendritic cells, monocytes, and macrophages. Several diseases are associated with changes in B cell subsets, and immunophenotyping by flow cytometry is often used to address the diagnosis and follow-up of patients during therapy [18].

B cells are identified as CD19-positive lymphocytes and can be further classified by flow cytometry into several subpopulations using different combinations of markers.

Of the 438 antibody clones analyzed, 147 bound to B cells in fWB. More in detail, about 45% and 65% of these clones caused a substantial change in cell frequency and antigen expression after cryopreservation (Table S5). Notably, 13 clones detectable in fWB became undetectable after cryopreservation in cPBMCs and nine in cWB.

**Figure 3:**
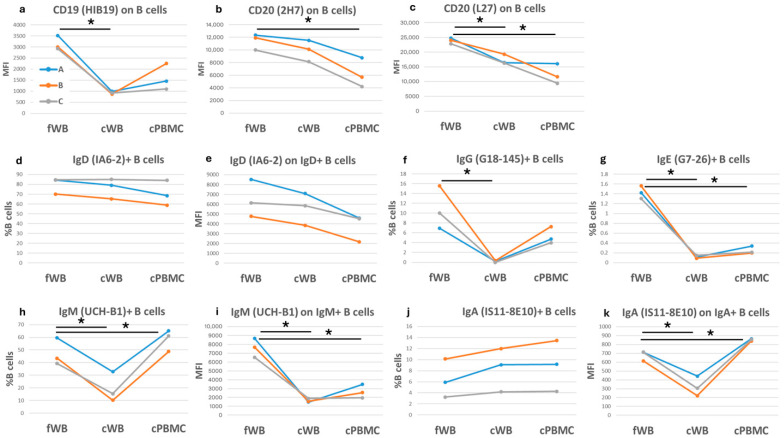
Comparison of B cells in fresh and corresponding cryopreserved samples. Each graph shows either the frequency of B cells positive for a specific antigen or the corresponding median fluorescence intensity (MFI) on the *y*-axis. The three colored lines (blue, orange, and grey) correspond to three blood samples, each of which was assessed under three different conditions: fresh (fWB), after blood cryopreservation (cWB), and after PBMC isolation and cryopreservation (cPBMC). These conditions are indicated on the *x*-axis. (**a**–**c**) CD19 and CD20 expression on B cells; (**d**–**k**) Immunoglobulins D, G, E, M, and A detection on B cells. A paired *t*-test was applied to calculate significance. * = *p*-values < 0.05.

The detection of CD19 expression, clone HIB19, a marker constitutively expressed by B lymphocytes, is reduced by 70% and 49% when measured on cWB and cPBMCs, respectively (Figure 3a). The CD20 antigen, a marker expressed by B cells except for plasma cells, was detected by the 2H7 and L27 clones. We observed a reduced detection of 36% in cWB only with clone L27, whereas the reduction was more pronounced (by 39% and 47% for clones 2H7 and L27, respectively) in cPBMCs compared to fWB (Figure 3b,c).

B cells are also characterized by the presence of the B cell receptor on their surface, which defines their antigen specificity, and by the expression of class II major histocompatibility complex (MHC) molecules (HLA-DR, -DP, and –DQ), which process exogenous peptides for presentation to CD4-positive helper T cells [19]. We found that after cryopreservation, the levels of HLA-DR, detected by clones L243 and G46-6, remained stable in cWB, whereas they were reduced by 38% in cPBMCs (Figure S3a,b).

Protein expression was much more variable after cryopreservation compared to the cell frequency. For example, CD21 expression, detected by clones B-Ly4 and 1048, was reduced by up to 42% in frozen samples, while the variation in the frequency of CD21-positive B cells was, on average, less than 2% (Figure S3c–f).

Among B cell markers typically used to characterize B cell maturation stages, we measured CD24, CD27 (L128 and M-T271), and CD38 (HB7 and HIT2). We observed that the frequency of positive cells remained more stable in cWB compared to cPBMCs. Instead, considering protein expression, both cPBMCs and cWB showed considerable differences compared to fresh samples, with changes often exceeding 30% (Figure S3g–p).

Among immunoglobulins, IgD-positive B cell frequency, ranging from 70 to 84% (mean 79.6%) in fresh samples, was stable after cryopreservation. Differently, IgD expression did not vary in cWB, but was reduced by 42% in cPBMCs (Figure 3d,e). The detection of the other immunoglobulins was much more affected by cryopreservation. For example, IgG-positive B cells were barely detectable in cWB and reduced by 49% in cPBMCs (Figure 3f). Similarly, IgE-positive B cells, which represent about 1.5% of total B cells, were hardly detectable in cryopreserved samples (Figure 3g). Conversely, IgM-positive and IgA-positive B cells were always detectable after freezing, although their expression varied significantly compared to fresh samples (Figure 3h–k).

### 2.4. Effect of Cryopreservation on T Cells

T lymphocytes are key players of adaptive immunity, mediating the cell-based immune response. T cells are characterized by the expression of the T cell receptor that allows them to recognize the MHC–antigen complex. We identified T cells as CD3-positive CD19-negative lymphocytes.

**Figure 4:**
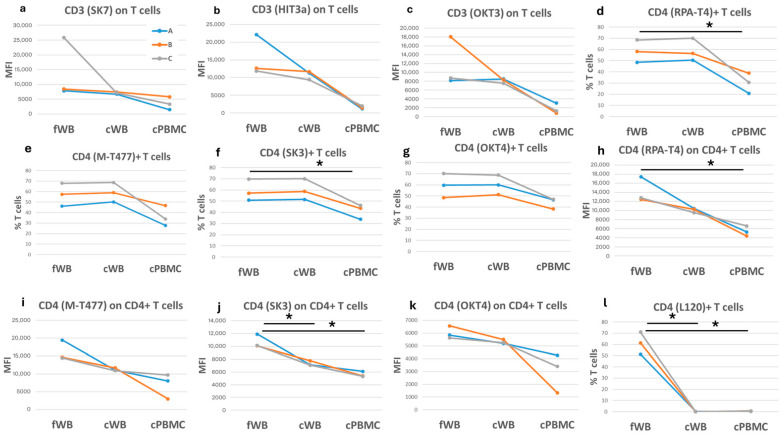
Comparison of T cells in fresh and corresponding cryopreserved samples. Each graph shows either the frequency of T cells positive for a specific antigen or the corresponding median fluorescence intensity (MFI) on the *y*-axis. The three colored lines (blue, orange, and grey) correspond to three blood samples, each of which was assessed under three different conditions: fresh (fWB), after blood cryopreservation (cWB), and after PBMC isolation and cryopreservation (cPBMC). These conditions are indicated on the *x*-axis. (**a**–**c**) CD3 expression on T cells; (**d**–**g**) frequency of CD4+ cells on T lymphocytes; (**h**–**l**) expression of CD4 on CD4+ T cells. A paired *t*-test was applied to calculate significance. * = *p*-values < 0.05.

Among the 438 antibodies tested in this study, 182 bound to T cells in fresh samples (Table S6). Approximately 42% of these clones caused a substantial change in the proportion of positive cells, while 70% caused substantial changes in antigen expression. Only two clones in cWB and three in cPBMCs were undetectable after cryopreservation. The detection of CD3 expression was reduced on T cells from cWB and, to a greater extent, in cPBMCs. This was particularly evident for the HIT3alpha clone, by which we observed a reduction in CD3 expression of 26% in cWB and 89% in cPBMCs, compared to fWB (Figure 4a–c).

The CD4 antigen, which characterizes helper T cells, was detected by five clones. Clones RPA-T4, M-T477, SK3, and OKT4 showed a reduction in CD4-positive T cell frequency only in cPBMCs (Figure 4d–g). Notably, the expression of CD4 was always reduced after cryopreservation, particularly in cPBMCs (Figure 4h–k). Differently, the L120 clone was detectable in fWB, but not after cryopreservation (Figure 4l).

Similarly, CD8 antigen, which characterizes cytotoxic T cells, was detected using five antibody clones. HIT8a and SK1 clones revealed an increase in the frequency of CD8-positive cells in cWB and a reduction in cPBMCs, compared to fresh samples (Figure S4a,b). However, the CD8 expression was consistently lower in both cWB and cPBMCs with all five clones (Figure S4c–g).

We also analyzed two markers that are typically used to identify regulatory T cells [20], such as the alpha chain of IL2 receptor (CD25, clone 2A3), and the subunit alpha of IL7 receptor (CD127, clone HIL-7R-M21). Clone 2A3 bound about 39% of T cells in fWB, but after cryopreservation, we observed a reduction of 27% and 69% of CD25-positive T cell frequency in cWB and cPBMCs, respectively (Figure S4h). When we considered CD25 expression, we observed a reduction by 38% in cWB and an increase by 50% in cPBMCs compared to fWB (Figure S4i).

After cryopreservation, clone HIL-7R-M21 identified a similar frequency of CD127-positive T cells in cryopreserved samples compared to fWB, while CD127 expression was reduced by 57% in cWB and 42% in cPBMCs (Figure S4j,k).

Furthermore, the frequency of CD27-positive T cells (clones M-T271 and L128) was stable in frozen samples (Figure S4l,m). However, the expression level of the memory marker CD27 was reduced after cryopreservation, and it was more reliable in cPBMCs compared to cWB (Figure S4n,o).

Importantly, we found that the two CD20 clones analyzed (L27 and 2H7) identified a subset of T cells that expressed CD20, although CD20 is a typical B cell marker. This rare population was recently described as having a pro-inflammatory function [21,22,23]. After cryopreservation, this population was strongly reduced in cPBMCs when using both CD20 clones (Figure S4p–s).

### 2.5. Effect of Cryopreservation on NKe Cells

Natural Killer (NK) cells are cytotoxic innate lymphoid cells involved in protection from pathogenic invasion and malignant cell transformation. We considered CD3-negative CD19-negative lymphocytes as NK-enriched (NKe) cells.

We found that 200 antibody clones (of the 438 tested) bound NKe cells. About 80% of these clones showed a variation of at least 20% in terms of cell frequency and antigen expression after cryopreservation. Notably, 3 and 9 markers were detectable in fWB, but undetectable after cryopreservation in cWB and cPBMCs, respectively (Table S7).

NK cells are usually classified by the different expression of the low-affinity IgG receptor CD16 and the neural cell adhesion molecule (NCAM) CD56 into: CD56bright/CD16neg and CD56dim/CD16pos [24,25,26].

**Figure 5:**
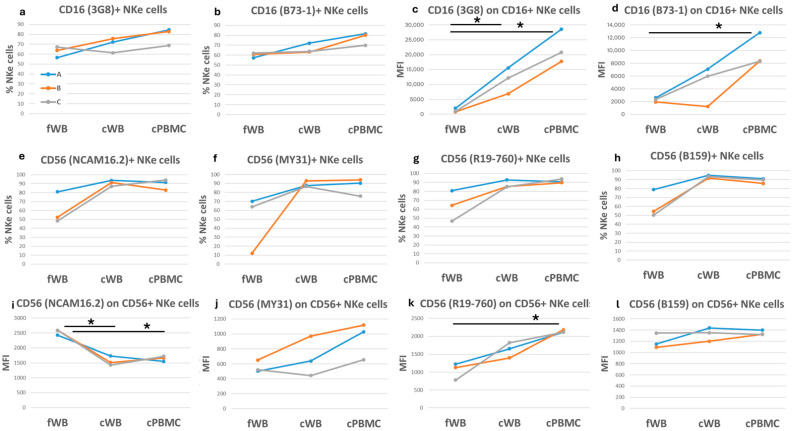
Comparison of NKe cells in fresh and corresponding cryopreserved samples. Each graph shows either the frequency of NKe cells positive for a specific antigen or the corresponding median fluorescence intensity (MFI) on the *y*-axis. The three colored lines (blue, orange, and grey) correspond to three blood samples, each of which was assessed under three different conditions: fresh (fWB), after blood cryopreservation (cWB), and after PBMC isolation and cryopreservation (cPBMC). These conditions are indicated on the *x*-axis. (**a**,**b**) Frequency of CD16+ cells on NKe lymphocytes; (**c**,**d**) CD16 expression on CD16+ NKe cells; (**e**–**h**) Frequency of CD56+ cells on NKe lymphocytes; (**i**–**l**) CD56 expression on CD56+ NKe cells. A paired *t*-test was applied to calculate significance. * = *p*-values < 0.05.

Both CD16 clones evaluated, 3G8 and B73-1, detected similar percentages of CD16-positive NKe cells in fWB. After cryopreservation, we observed weak variations in cWB and an increase in CD16-positive NKe cell frequency in cPBMCs (27% and 29%, for clones 3G8 and B73-1, respectively) (Figure 5a,b). However, CD16 expression on average increased after cryopreservation, especially in cPBMCs (Figure 5c,d).

The CD56 molecule was recognized by four clones (NCAM16.2, MY31, R19-760, and B159), which bound to 49–64% of total NKe cells in fWB. After cryopreservation, the frequency of CD56-positive cells increased, on average, by over 40% in both cWB and cPBMCs (Figure 5e–h). The NCAM16.2 clone detected a reduction in the CD56 expression levels, whereas MY31 and R19-769 clones showed an increase, and the B159 clone showed no substantial variation (Figure 5i–l).

### 2.6. Effect of Cryopreservation on Monocytes

Monocytes are myeloid cell members of the mononuclear phagocyte system representing 5–10% of the total leukocyte cells in human peripheral blood [27].

The known loss of granules following granulocyte cryopreservation made it difficult to distinguish monocytes from granulocytes morphologically in cWB. We thus evaluated the effect of cryopreservation on monocyte markers only on cPBMCs, in which granulocytes were removed before freezing samples.

Among the 438 antibodies we tested, 196 bound monocytes in fWB. About 33% and 83% of these clones detected a variation of at least 20% in terms of cell frequency and antigen expression following cryopreservation, respectively (Table S8).

**Figure 6:**
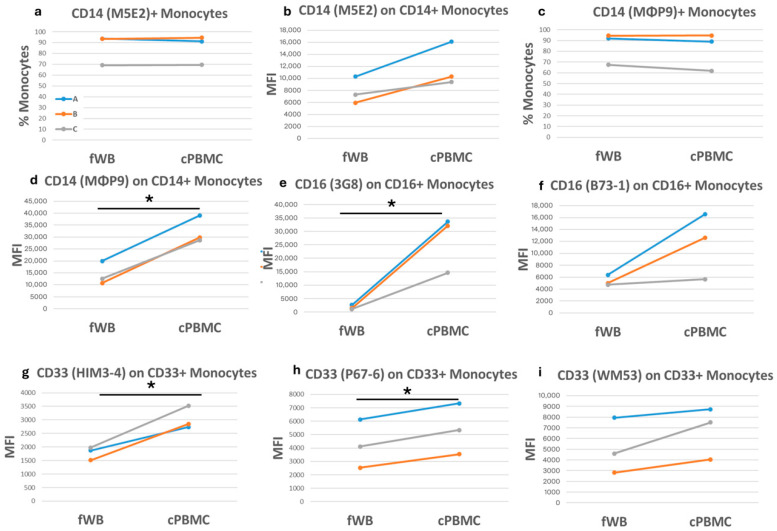
Comparison of monocytes in fresh and corresponding cryopreserved samples. Each graph shows either the frequency of monocytes positive for a specific antigen or the corresponding median fluorescence intensity (MFI) on the *y*-axis. The three colored lines (blue, orange, and grey) correspond to three blood samples, each of which was assessed under three different conditions: fresh (fWB), after blood cryopreservation (cWB), and after PBMC isolation and cryopreservation (cPBMC). These conditions are indicated on the *x*-axis. (**a**–**d**) Detection of CD14 antigen on CD14+ monocytes; (**e**,**f**) CD16 expression on CD16+ monocytes; (**g**–**i**) CD33 expression on CD33+ monocytes. A paired *t*-test was applied to calculate significance. * = *p*-values < 0.05.

CD14 and CD16 molecules are usually used to classify monocytes into three subclasses (classical, non-classical, and intermediate) [28,29]. Among the antibodies we tested, clones M5E2 and MΦP9 recognized the CD14 molecule on about 85% of monocytes, while clones 3G8 and B-73-1 detected the CD16 antigen on about 15% of monocytes in fWB. After cryopreservation, the frequencies of CD14-positive monocytes in cPBMCs were, on average, similar to those in fWB, whereas the expression levels of both CD14 and CD16 were augmented (Figure 6a–f). Similarly, clones HIM3-4, P67-6, and WM53, which recognize CD33, showed no substantial variation in positive cell frequency after cryopreservation, but an increase of about 30% in CD33 expression (Figure 6g–i).

Substantial changes also occur for markers generally used for monocyte characterization, such as HLA-DR, CD11b, CD11c, and CD32 (Figure S5a–h). Differently, the expression level of the adhesion molecule CD321, recognized by the M-Ab-F11 clone, was stable after freezing (Figure S5i).

### 2.7. Chemokine Receptor Staining in Fresh Blood at Different Temperatures

Previous studies showed that chemokine receptors are difficult to detect due to their peculiar conformation and flexibility [30]. To improve their binding, we incubated 17 antibodies specific for chemokine receptors both at room temperature (RT) and at 37 °C. We observed that the staining temperature had a considerable impact on detecting the expression of most of these receptors. Specifically, nine of the 17 antibodies exhibited an increased expression by at least 20% when the samples were stained at 37 °C compared to those stained at RT. Notably, the frequency of positive cells is less affected by staining conditions (Tables S9–S12).

**Figure 7:**
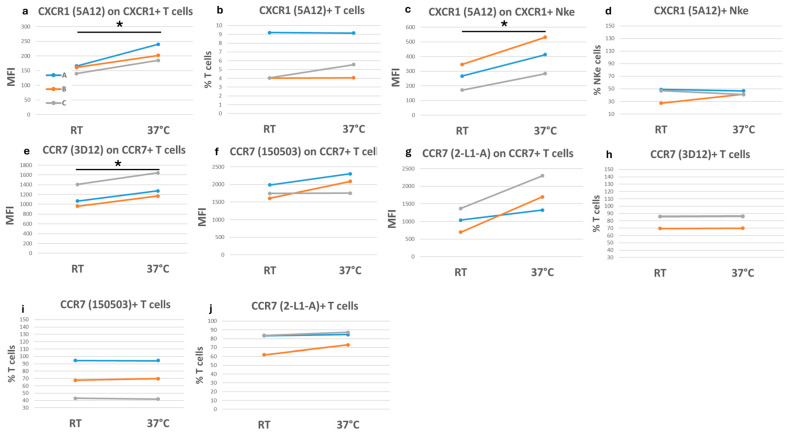
Chemokine receptor staining in fresh blood at different temperatures. Each graph shows either the frequency of different cell subsets positive for a specific chemokine receptor or the corresponding median fluorescence intensity (MFI) as indicated on the *y*-axis. The three colored lines (blue, orange, and grey) correspond to three fresh blood samples, each of which was assessed by performing the staining protocol at room temperature (RT) or at 37 °C. These conditions are indicated on the *x*-axis. (**a**–**d**) CXCR1 detection on lymphocyte subsets; (**e**–**j**) CCR7 detection on T cells. A paired *t*-test was applied to calculate significance. * = *p*-values < 0.05.

For example, the expression of CXCR1, detected by the antibody clone 5A12, increased by 34% on T lymphocytes and by 58% in NKe cells when samples were stained at 37 °C. However, the frequency of CXCR1-positive cells was stable between the two staining conditions (Figure 7a–d; Tables S9 and S10).

CCR7 is expressed by B cells, mature dendritic cells, and by several T-cell subpopulations, including naive, regulatory, and central memory T cells [31]. Frequently, CCR7 is used in combination with CD45RA to identify maturation stages of T cells [32]. Here, we analyzed three CCR7-specific human clones, 3D12, 150503, and 2-L1-A. We observed that, unlike 150503 and 2-L1-A clones, the 3D12 clone did not bind B cells (Table S11). However, all clones recognized CCR7 on a subset of T cells. In more detail, the 3D12, 150503, and 2-L1-A clones increased the detection of CCR7 expression on T cells of about 19%, 16%, and 80%, respectively, when stained at 37 °C compared with RT. Nevertheless, staining temperature never affected the frequency of the corresponding CCR7-positive T cells (Figure 7e–j).

For other markers, such as CXCR3 and CCR6, the antibody binding was weakly affected by the staining temperature, resulting in a similar frequency and antigen expression at 37 °C and RT (Tables S9 and S11).

### 2.8. Comparison of Chemokine Receptors in Cryopreserved and Fresh Samples

Because we generally found a higher level of chemokine receptor expression at 37 °C (than RT) in fresh samples, we used this temperature to stain frozen samples. We then compared them with the corresponding fresh samples assessed at the same temperature.

We observed that the detection of chemokine receptors is differently affected by cryopreservation (Tables S13–S16).

**Figure 8:**
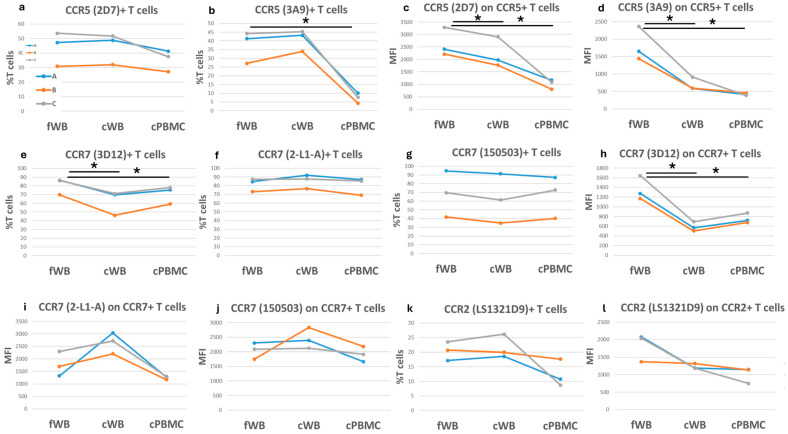
Comparison of chemokine receptors in fresh and corresponding cryopreserved samples.Each graph shows either the frequency of different cell subsets positive for a specific chemokine receptor or the corresponding median fluorescence intensity (MFI) as indicated on the *y*-axis. The three colored lines (blue, orange, and grey) correspond to three blood samples, each of which was assessed in three different conditions: fresh (fWB), after blood cryopreservation (cWB), and after PBMC isolation and cryopreservation (cPBMC). These conditions are indicated on the *x*-axis. (**a**,**b**) Frequency of CCR5+ cells on T lymphocytes; (**c**,**d**) CCR5 expression on CCR5+ T cells; (**e**–**g**) Frequency of CCR7+ cells on T lymphocytes; (**h**–**j**) CCR7 expression on CCR7+ T cells; (**k**,**l**) CCR2 detection on T cells. A paired *t*-test was applied to calculate significance. * = *p*-values < 0.05.

For example, the CCR5 antigen was identified by the 2D7 and 3A9 antibody clones. In cWB, both clones detected CCR5-positive T cell proportions similar to fWB, while both clones showed a reduction (17% with 2D7 and 61% with 3A9) in antigen expression (Figure 8a–d; Table S13). In cPBMCs, no substantial variation was observed in CCR5-positive T cell proportions using the 2D7 clone, while 3A9 determined a reduction of 81%. Furthermore, a strong reduction in CCR5 expression was observed on positive T cells from cPBMCs (61% with 2D7 and 76% with 3A9) compared to fresh samples.

Another example is represented by CCR7, of which we analyzed the 2-L1-A, 150503, and 3D12 clones. In frozen samples, clones 2-L1-A and 150503 showed no variations in the CCR7-positive B cell proportions, whereas the 3D12 clone failed to bind CCR7, in line with what we observed in fresh blood (Table S14). When we considered T cells, only the 3D12 clone showed a variation in CCR7-positive T cell frequency higher than 20% in cWB, while it was milder in cPBMCs (13%). After cryopreservation, no substantial variation was observed in cell frequency using both 2-L1-A and 150503 clones (Figure 8e–g).

However, antigen expression detected with clone 3D12 was always reduced after cryopreservation of 57% in cWB and 44% in cPBMCs (Figure 8h). Clone 2-L1-A exhibited variations in CCR7 expression higher than 20% with an increase in cWB and a decrease in cPBMCs, whereas clone 150503 detected milder variations in cryopreserved samples (Figure 8i,j).

Furthermore, we found that the CCR2 clone LS1321D9 showed a reduction in CCR2-positive T cell frequency only in cPBMCs and a general reduction in antigen expression on T cells after cryopreservation (Figure 8k,l). Interestingly, CCR2-specific clone 48607 failed to detect CCR2-positive T cells after cryopreservation (Figure S6a).

The CXCR3 clone 1C6 always detected a reduction in antigen expression and CXCR3-positive T cell frequency after cryopreservation, which is much more evident in cWB (Figure S6b,c).

## 3. Discussion

Cryopreserved samples are widely used in large-scale immunophenotyping studies in which blood collection and immunophenotyping take place in geographically separated sites, and to reduce “batch” variability arising from different operators, instruments, or reagents. Freezing and thawing procedures can damage cells by ice crystal formation, which compromises cell membrane integrity, and by cryoprotectants, such as dimethyl sulfoxide, which is a cell toxic agent. As a result, cell injuries can affect the expression of specific antigens on the cell surface and overall cell functionality [33,34]. Thus, the comparison of immunophenotyping in fresh and cryopreserved samples is essential to develop the most appropriate freezing and thawing protocols and select the most reliable markers to be tested after cryopreservation.

Our study systematically measured 438 surface antibody clones on freshly stained samples (blood) and evaluated whether these markers were also detectable in cryopreserved blood and lympho-monocytes. To support scientists in designing cytofluorimetric panels, we shared the staining of each clone tested in an interactive database available online (http://flowcloc.irgb.cnr.it/, accessed on 6 February 2026), namely FlowCLOc. Overall, our results showed that the detection of 283 and 262 out of 438 antibodies tested was affected by cryopreservation in cPBMCs and in cWB, respectively, in terms of frequency of the corresponding positive cells and, more frequently, intensity of protein expression. NKe cells showed the highest percentage of alteration, exceeding 80%.

Our data are in line with the reduced recovery of B cells [35], regulatory T cells [12,36], and reduced functionality of NK observed in previous studies [37], with the substantial addition of evaluating the level of thousands of antigens in these broadly classified cell types.

Indeed, we observed a reduction in both CD4-positive and CD25-positive T cell frequency in cPBMCs, which is in line with the reduced recovery and functionality of regulatory T cells observed after cryopreservation [36,38,39]. Similarly, we observed high variations in many antigens expressed on NKe cell surface, consistent with the impaired cytotoxic activity described for cryopreserved NK cells [40,41,42].

We also observed markers whose detection after freezing was different compared to previous studies. For example, a previous work of Mark and collaborators [41] described a decrease in the CD16-positive subpopulation of NK cells after cryopreservation of PBMCs, whereas we observed their increase in both frequency and expression level. This is probably due to membrane damage, which causes a nonspecific higher antigen–antibody interaction.

Similarly, the expression of other markers, such as CD47 and CD58 on NKe cells or CD32 on monocytes, also increased after cryopreservation.

Although cWB is, on average, more similar to fresh blood in terms of cell frequency and antigen expression levels, cPBMCs are generally considered a more stable starting point than frozen blood. In fact, granulocytes are susceptible to degradation following freezing; thus, the presence of a significant percentage of partially degraded granulocytes after freezing and the consequent release of intracellular material could cause nonspecific lymphocyte-monocyte activation [43,44]. Thus, careful attention should be taken before selecting the most appropriate cryopreserved material for subsequent experiments.

The present work could be very useful in terms of technological advances, providing new tools for choosing the most suitable reagents and conditions for setting up informative experiments, both for functional and genetic small- and large-scale studies. However, it also has some limitations. For instance, the small sample size used to analyze each clone needs to be enlarged. Indeed, a larger sample size would allow for more robust statistical analyses and help ensure that the conclusions drawn are unequivocal, as tests based on small samples can be misleading. Furthermore, the use of markers capable of specifically identifying monocytes and granulocytes in the backbone panel would also allow us to evaluate the tested antibodies in the myeloid compartment. Indeed, we observed that granulocytes are the leukocyte subpopulation most affected by cryopreservation. After freezing, granulocytes changed their morphology [12,13,45], probably due to granule release and cell death [46]. Moreover, the reduced intracellular complexity of granulocytes in cWB samples precludes their discrimination from monocytes by morphological parameters. Thus, including monocytic- and granulocyte-specific markers (such as CD14 and CD15, respectively) in the antibody backbone would allow us to accurately measure these cell populations in cryopreserved samples.

Moreover, 108 markers of the 438 analyzed (24%) were barely or not detected in fresh samples (Table S17), likely because (i) the population expressing them is rare, (ii) these markers are only detectable after activation, but we are evaluating the baseline status, and (iii) the antibody is not binding properly to the cognate antigen.

Further studies on the expression of intracellular markers, as well as comparison between basal and stimulated cell conditions, before and after freezing, could provide a broader and more precise view of the effect of cryopreservation on immune cell phenotype and function.

## 4. Materials and Methods

### 4.1. Samples

Whole blood samples were collected from healthy donors belonging to the SardiNIA project cohort [47] into vacutainers containing sodium heparin (BD Life Sciences Biosciences, Franklin Lakes, NJ, USA, #367878) between 8:00 a.m. and 9:00 a.m. Samples were processed within two hours after collection and maintained at room temperature until processing or cryopreservation as described below.

All participants signed informed consent to study protocols approved by the Sardinian Regional Ethics Committee (prot. n. 2171/CE).

### 4.2. Cryopreservation of Whole Blood

One volume of fWB was mixed with one volume of a freezing mix consisting of 20% dimethyl sulfoxide (DMSO; Sigma Aldrich, St. Louis, MO, USA #D-2650) in RPMI 1640 medium (Lonza, Walkersville, MD, USA, #BE12-167, containing glucose at a concentration of 2 g/L), resulting in a final DMSO concentration of 10%. Samples were subjected to controlled-rate freezing (−1 °C/min) using a dedicated device (Nalgene Mr. Frosty, Thermo Fischer Scientific, Waltham, MA, USA, #5100-0001), and then stored at −80 °C until use.

### 4.3. Separation and Storage of Peripheral Blood Mononuclear Cells

PBMCs were isolated using BD Vacutainer Mononuclear Cell Preparation Tube (CPT) technology (BD Life Sciences Biosciences, Franklin Lakes, NJ, USA, #362780), following the manufacturer’s instructions. Briefly, whole blood was drawn directly into the CPT and centrifuged at RT. The PBMC layer was aspirated and washed once with Phosphate Buffered Saline (PBS) 1×X, and a second time with full RPMI 1640 medium (supplemented with 10% of Fetal Bovine Serum (FBS), Sodium Pyruvate, and Glutamine). PBMCs resuspended in full RPMI were diluted with an equal volume of freezing media consisting of FBS with 20% DMSO (final concentration of 10%). Samples were stored at −80 °C using a Mr. Frosty container and then transferred to a −150 °C freezer until their use.

All cryopreserved samples (both cPBMCs and cWB) were frozen for approximately 18 months.

### 4.4. Cell Staining

#### 4.4.1. Fresh Whole Blood

An amount of 300 μL of fWB was stained with an antibody backbone (Table S18) and 438 antibodies conjugated with the BV421 fluorochrome (Table S19) for 15 min at RT in the dark.

To assess the purity of the NK-enriched cells, a Lyse-No-wash protocol was applied as previously described [1]. Lysed cells were stained with the 6-color TBNK kit from BD Biosciences (cat. 644611).

#### 4.4.2. Chemokine Receptor Staining

An amount of 300 μL of stained fWB was incubated for 15 min in two modalities: at RT or at 37 °C.

After incubation, all samples were washed with Stain Buffer (BD cat. 554657), lysed with BD FACS Lysing solution (cat. 349202) for 10 min in the dark, and then washed with Stain Buffer.

#### 4.4.3. Immunoglobulin Staining

An amount of 300 μL of fWB was lysed twice with BD Pharm Lyse solution (cat. 555899), and then washed with Stain Buffer. Cells were stained with the antibody backbone and the anti-immunoglobulin antibody for 30 min at RT in the dark. After incubation, samples were washed with PBS 1× and analyzed.

#### 4.4.4. Cryopreserved Whole Blood and PBMCs

Cryopreserved whole blood and PBMC samples were quickly thawed at 37 °C and washed with PBS 1× to remove any residual DMSO. Cells were then incubated with the antibody backbone (Table S18), the BV421-conjugated clone (Table S19), and the Fixable Viability Stain 780 (FVS780, BD Biosciences, San Jose, CA, USA, #565388) for 15 min at RT in the dark.

After incubation, only whole blood samples were lysed with BD FACS lysing solution to remove red cells.

Samples were finally washed with PBS 1× and acquired by BD FACS CANTO II analyzer (BD Biosciences). The cytometer configuration is reported in Table S20.

Cytometer settings, reproducibility of measurements, and calculation of compensation were performed as previously described [1]. Flow cytometric data were analyzed manually by BD FACSDiva^TM^ software (BD Biosciences, version 9.0).

For each condition (fWB, cPBMCs, and cWB), each antibody clone was measured on three samples by evaluating median fluorescence intensity and, when appropriate, cell frequency with respect to hierarchically higher cell populations. When an antigen was characterized by a bimodal distribution, its median fluorescence intensity was evaluated on positive cells only.

### 4.5. Statistical Analysis

Cell frequency and median fluorescence intensity in frozen samples were compared to those in fresh blood by using the following ratios: cPBMCs/fWB and cWB/fWB. A ratio greater than 1 indicates a trait increase in the cryopreserved sample compared to the corresponding fresh one. Conversely, a ratio lower than 1 indicates a trait reduction in the cryopreserved samples. When the ratio is equal to 1, the trait mean is the same, indicating no difference in frozen and fresh samples. In the Section 2, this ratio is expressed as a percentage of increase or decrease.

The effect of staining temperature on chemokine receptors was evaluated by comparing each sample stained at 37 °C with the corresponding sample stained at RT. The trait ratio 37 °C/RT was calculated, similarly to the cryopreserved/fresh ratio.

All results are reported as means of the described ratios. The difference observed in frozen vs. fresh samples (or 37 °C vs. RT conditions) was considered substantial when it was higher than 20%, which corresponds to mean ratios >1.2 or <0.8.

A paired *t*-test was performed to determine whether there was a statistically significant difference in variation between the fresh and frozen samples (cPBMCs and cWB) or between the staining conditions at RT and 37 °C. *p*-values below 0.05 were considered to be statistically significant.

### 4.6. FlowCLOc Generation

The FlowCLOc platform was developed in React (https://react.dev/, accessed on 6 February 2026; version 18.3.1) with TypeScript (https://www.typescriptlang.org/, accessed on 6 February 2026; version 5.6.2) and bundled with Vite (https://vitejs.dev/, accessed on 6 February 2026; version 5.4.0). The static bundle is served by Nginx (https://nginx.org/, accessed on 6 February 2026; version 1.27-alphine) within a Docker container (https://www.docker.com/, accessed on 6 February 2026, version 28.2.2) and Docker Compose (https://docs.docker.com/compose/, accessed on 6 February 2026, version 2.40.0). FlowCLOc displays cytometry panels in PNG format indexed by a static file and provides cascading filters with incremental search functionality, namely, antigen, clone, sample, temperature, and cell/condition.

## Data Availability

The original contributions presented in this study are included in the article/Supplementary Materials. Further inquiries can be directed to the corresponding author.

## Supplementary Materials

The following supporting information can be downloaded at: https://www.mdpi.com/article/10.3390/ijms27041664/s1.
