# Supplementary material for: FlowCLOc, a New Tool for Selecting the Most Appropriate Antibodies in Flow Cytometry
Source: Int J Mol Sci. 2026 Feb 9;27(4):1664. doi: 10.3390/ijms27041664 (PMC12940311; doi:10.3390/ijms27041664)
Supplement: Supplementary file 1 [file ijms-27-01664-s001.zip › Supplementary Figure captions.pdf]

Supplementary Figure legends

Figure S1. Gating strategy used to compare NKe and NK cells. Dot plots showing A) Lymphocytes (pink) gated by the morphological parameter SSC-A and CD45 positivity, B) Lymphocytes divided in CD3+ (T cells, green) and CD3- (black). Within CD3- subset, identification of C) Nke cells (orange) as negative for both CD3 and CD19 and D) NK cells (light blue) as positive for CD16 and/or CD56.

Figure S2. Representative example of detection of viable cells in a thawed sample. Dot plot showing A. single cells (red); b. Morphologically identified lymphocytes (light green) and monocytes (light blue); c. lymphocytes that are negative for the fixable viability stain (FVS780) are considered viable (dark green); d. monocytes that are FVS78-negative are considered viable (blue).

Figure S3. Effect of cryopreservation on B cells. The graphs compare B cells in fresh and corresponding cryopreserved samples. Each graph shows either the frequency of B cells positive for a specific antigen or the corresponding median fluorescence intensity (MFI) on the y-axis. The three colored lines (blue, orange, and grey) correspond to three blood samples, each of which was assessed under three different conditions: fresh (fWB), after blood cryopreservation (cWB), and after PBMC isolation and cryopreservation (cPBMC). These conditions are indicated on the x-axis. A paired T-test was applied to calculate significance. *=P-values<0.05

Figure S4. Effect of cryopreservation on T cells. The graphs compare T cells in fresh and corresponding cryopreserved samples. Each graph shows either the frequency of T cells positive for a specific antigen or the corresponding median fluorescence intensity (MFI) on the y-axis. The three colored lines (blue, orange, and grey) correspond to three blood samples, each of which was assessed under three different conditions: fresh (fWB), after blood cryopreservation (cWB), and after PBMC isolation and cryopreservation (cPBMC). These conditions are indicated on the x-axis. A paired T-test was applied to calculate significance. *=P-values<0.05.

Figure S5: Effect of cryopreservation on Monocytes. The graphs compare monocytes in fresh and corresponding cryopreserved samples. Each graph shows either the frequency of monocytes positive for a specific antigen or the corresponding median fluorescence intensity (MFI) on the y-axis. The three colored lines (blue, orange, and grey) corre-spond to three blood samples, each of which was assessed under three different conditions: fresh (fWB), after blood cryopreservation (cWB), and after PBMC isolation and cryopreservation (cPBMC). These conditions are indicated on the x-axis. A paired T-test was applied to calculate significance. *=P-values<0.05.

Figure S6: Effect of cryopreservation on chemokine receptors. The graphs comparise chemokine receptors in fresh and corresponding cryopreserved sam-ples. Each graph shows either the frequency of different cell subsets positive for a specific chemokine receptor or the corresponding median fluorescence intensity (MFI) as indicated on the y-axis. The three colored lines (blue, orange, and grey) correspond to three blood samples, each of which was assessed in three different conditions: fresh (fWB), after blood cryopreservation (cWB), and after PBMC isolation and cryopreservation (cPBMC). These conditions are indicated on the x-axis. A paired T-test was applied to calculate significance. *=P-values<0.05.
