# Supplementary figures and images for: FlowCLOc, a New Tool for Selecting the Most Appropriate Antibodies in Flow Cytometry
Source: Int J Mol Sci. 2026 Feb 9;27(4):1664. doi: 10.3390/ijms27041664 (PMC12940311; doi:10.3390/ijms27041664)

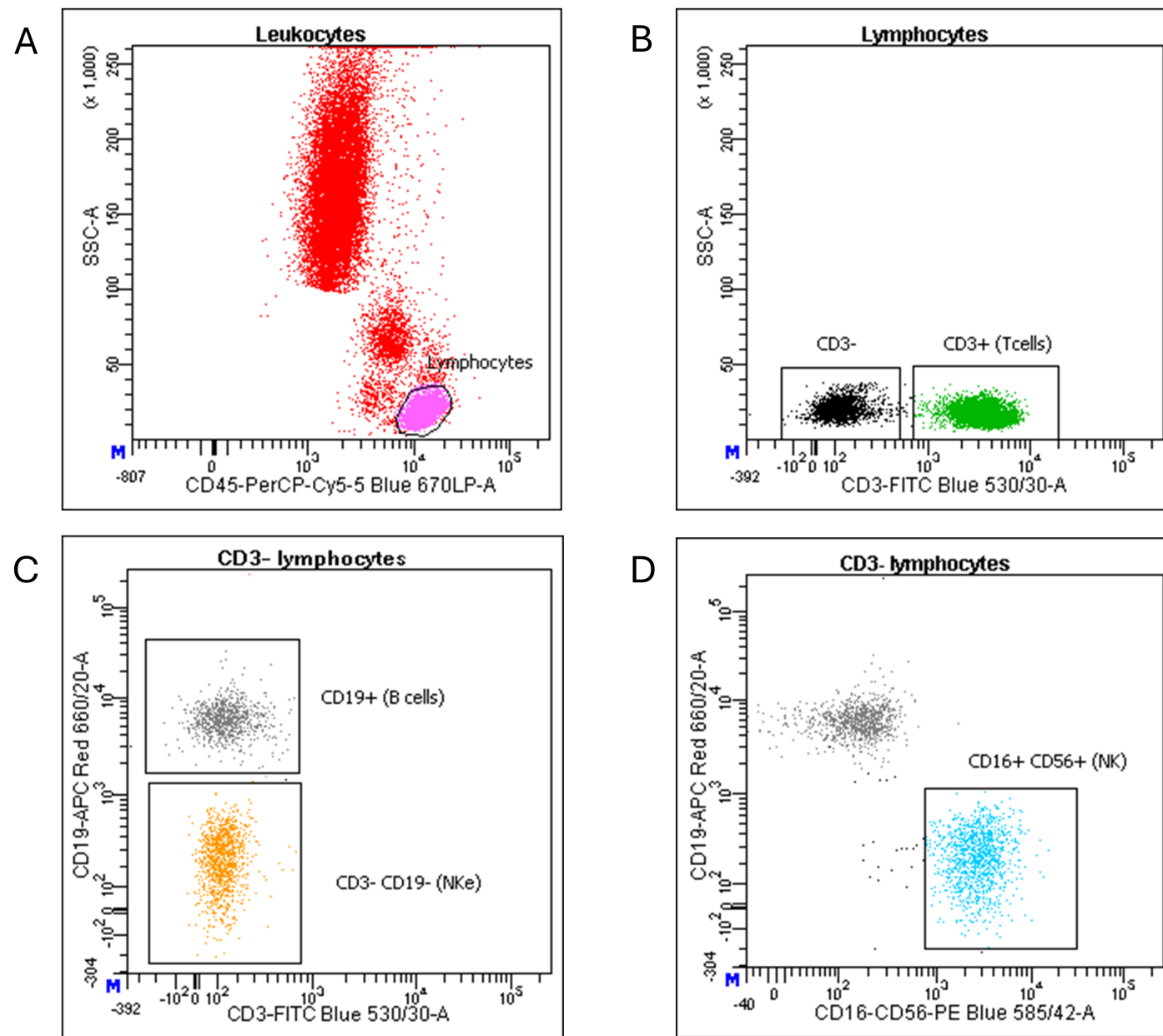

**Supplementary Figure S1**

Supplement: Supplementary file 1 [file ijms-27-01664-s001.zip › Figure S1.pdf]

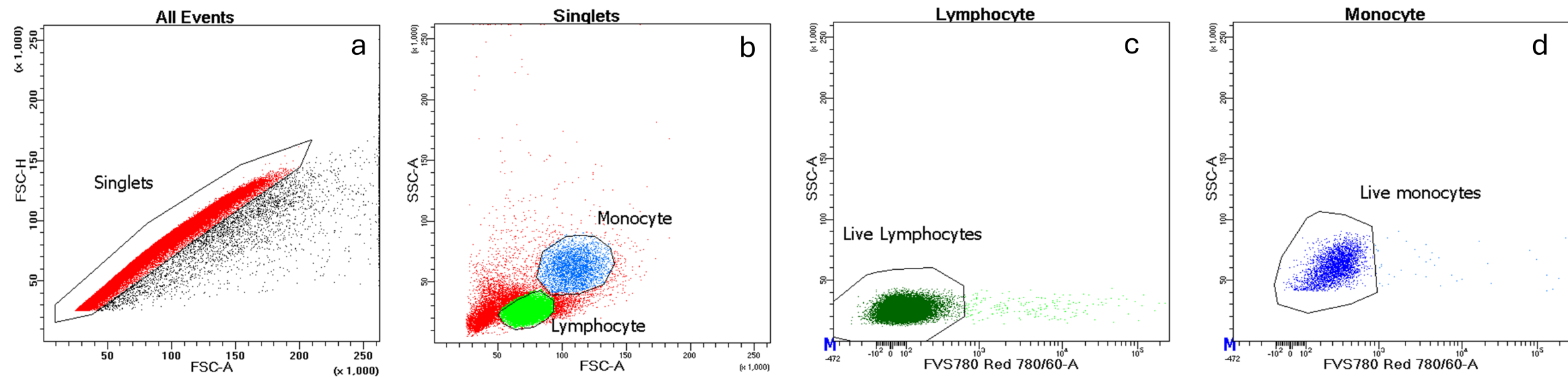

**Supplementary Figure S2**

Supplement: Supplementary file 1 [file ijms-27-01664-s001.zip › Figure S2.pdf]

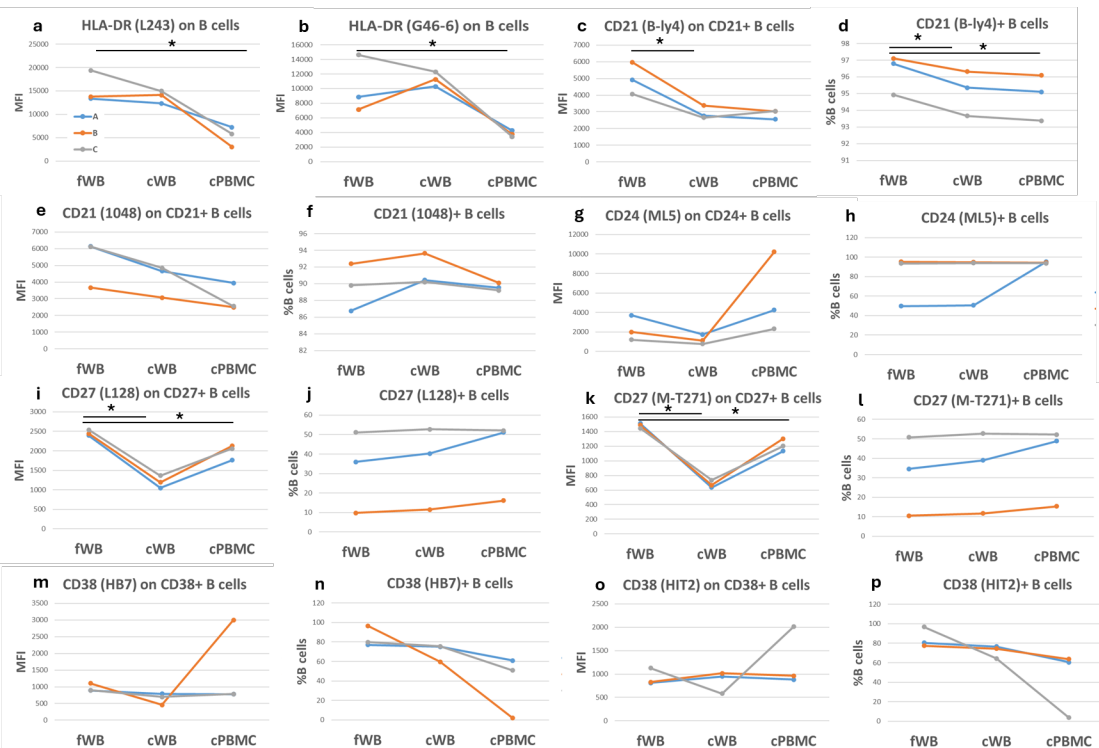

Supplementary Figure S3

Supplement: Supplementary file 1 [file ijms-27-01664-s001.zip › Figure S3.pdf]

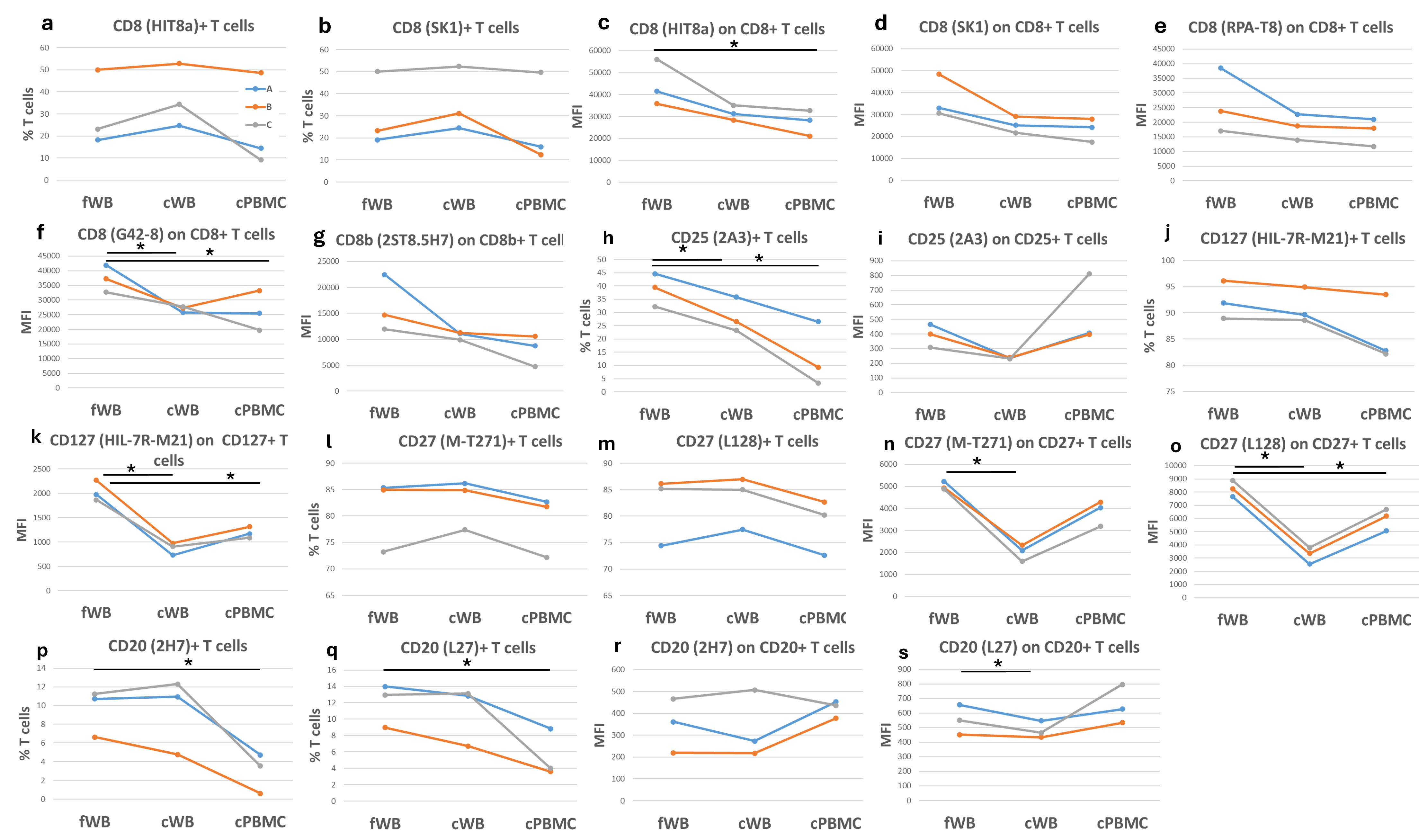

Supplement: Supplementary file 1 [file ijms-27-01664-s001.zip › Figure S4.png]

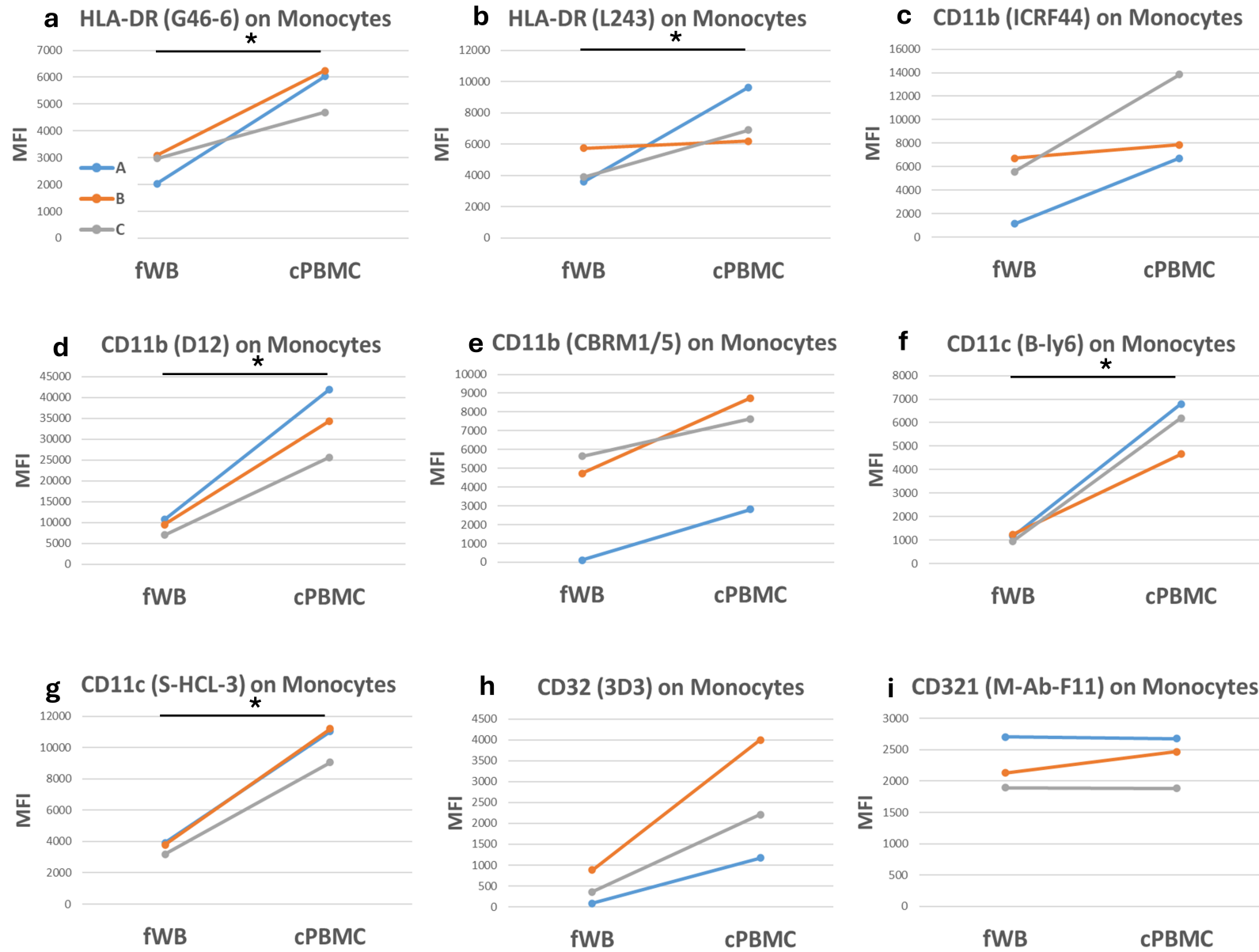

Supplementary Figure S5

Supplement: Supplementary file 1 [file ijms-27-01664-s001.zip › Figure S5.pdf]

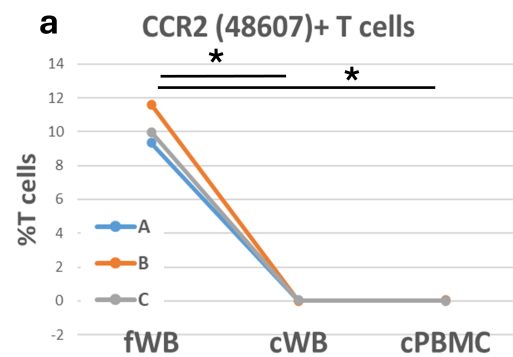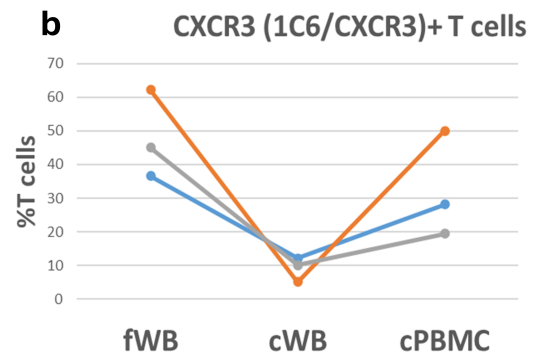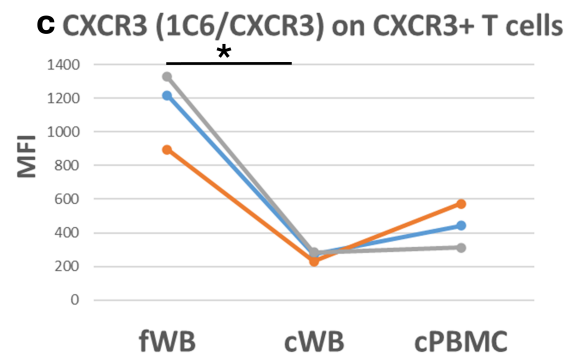

**Supplementary Figure S6**

Supplement: Supplementary file 1 [file ijms-27-01664-s001.zip › Figure S6.pdf]
